# Multicenter Prospective Validation of an Updated Proprietary Sepsis Prediction Model

**DOI:** 10.1001/jamanetworkopen.2026.0181

**Published:** 2026-02-27

**Authors:** Andrew Wong, Danielle Currey, Megan Schwinne, Brenna Park-Egan, Sean Meyer, Andrew Gutting, Jie Cao, Sharaf Khan, Raymund Dantes, Tony Pan, Timothy Buchman, Karandeep Singh, Sivasubramanium V. Bhavani, Patrick G. Lyons, Michael W. Sjoding, Yasir Tarabichi

**Affiliations:** 1 University of Michigan Medical School, Ann Arbor; 2 Case Western Reserve University School of Medicine, Cleveland, Ohio; 3 Center for Clinical Informatics Research and Education, MetroHealth, Cleveland, Ohio; 4 Department of Biomedical Informatics, Emory University School of Medicine, Atlanta, Georgia; 5 Division of Pulmonary, Allergy, and Critical Care Medicine, Oregon Health & Science University, Portland; 6 Joan and Irwin Jacobs Center for Health Innovation, UC San Diego Health, San Diego, California; 7 Division of Biomedical Informatics, Department of Medicine, University of California, San Diego; 8 Department of Emergency Medicine, Emory University School of Medicine, Atlanta, Georgia; 9 Department of Medicine, Emory University School of Medicine, Atlanta, Georgia; 10 Department of Surgery, Emory University School of Medicine, Atlanta, Georgia; 11 Critical Care Center, Emory University School of Medicine, Atlanta, Georgia; 12 Department of Pulmonary and Critical Care Medicine, MetroHealth, Cleveland, Ohio

## Abstract

**Importance:**

The Epic Sepsis Model version 2 (ESM v2) is a widely implemented proprietary sepsis prediction model, but no multicenter, external validation of its performance has been reported to guide adoption and use.

**Objective:**

To conduct a multicenter validation of the ESM v2 to compare performance against the original ESM v1, outline differences across heterogenous clinical sites, and compare model performance against clinician recognition of sepsis.

**Design, Setting, and Participants:**

This prognostic study included adult inpatient encounters at 4 large US health systems between August 31, 2023, and March 11, 2025. At each site, data were collected for a consecutive period immediately following new model implementation. Data were analyzed from July 23 to August 19, 2025.

**Main Outcomes and Measures:**

Sepsis was defined using Sepsis-3 clinical consensus criteria. Model discrimination was assessed using area under the receiver operating characteristic curve (AUROC) at the encounter level and prediction level with 4-hour, 12-hour, and hospitalization-wide time horizons. Performance against clinician recognition of sepsis was measured using antibiotics, lactate, and body culture orders.

**Results:**

Of 227 091 inpatient encounters, 7401 (3.3%; median [IQR] age, 65 [54-75] years; 3359 [45.4%] female; 2.7% Asian; 24.6% Black; 64.6% White; 7.1% Hispanic ethnicity) met sepsis criteria and 219 690 (96.7%; median [IQR] age, 48 [33-65] years; 123 563 [56.2%] female; 2.5% Asian; 38.8% Black; 49.6% White; 9.6% Hispanic ethnicity) did not. At the encounter level, the AUROC ranged from 0.82 (95% CI, 0.81-0.83) to 0.92 (95% CI, 0.92-0.93) across study sites. At the prediction level with a 12-hour time horizon, the AUROC ranged from 0.75 (95% CI, 0.74-0.75) to 0.85 (95% CI, 0.85-0.85). Comparison against clinician recognition of sepsis yielded a minor decrease in performance, with the resulting encounter-level AUROC ranging from 0.80 (95% CI, 0.79-0.81) to 0.90 (95% CI, 0.89-0.90) across sites. Positive predictive values remained low, from 0.13 (95% CI, 0.13-0.14) to 0.26 (95% CI, 0.25-0.27), with a high number needed to evaluate and high alert burden.

**Conclusions and Relevance:**

In this prognostic study of a new sepsis prediction model, a multicenter prospective validation performed across 4 major US health systems found improved discrimination for the early prediction of sepsis but noted high institutional variability, low positive predictive value, and high alert burden.

## Key Points

**Question:**

How accurate is the Epic Sepsis Model version 2, an updated proprietary sepsis prediction model implemented at hundreds of US hospitals, at predicting sepsis?

**Findings:**

This prognostic study of 227 091 inpatient encounters across 4 major US health systems found that the model had an area under the receiver operating curve between 0.82 and 0.92 but demonstrated high institutional variability, low positive predictive value, and high alert burden.

**Meaning:**

These findings suggest that institutions implementing this model should conduct local validation studies to verify performance, integrate clinical workflows to manage false positives, and implement alert silencing strategies to reduce alert burden.

## Teaser

This prognostic study compares the performance of Epic Sepsis Model version 2 against the original Epic Sepsis Model and against clinician recognition across 4 major US health systems.

## Introduction

Sepsis is a leading cause of inpatient mortality in the US,^1,2,3^ and early identification and treatment of sepsis can improve outcomes and save lives.^4,5^ Screening for sepsis among hospitalized patients is strongly recommended by the Surviving Sepsis Campaign,^6^ which has resulted in broad adoption of automated sepsis prediction tools across US hospitals.^7^ However, such tools often lack generalizability and are commonly implemented by health systems without internal validation,^8^ potentially compromising sepsis care through missed or delayed treatment. Multicenter external validation studies evaluate model performance and generalizability and provide guidance for the safe and effective adoption of clinical prediction models.^9,10,11^

In 2018, Epic Systems released the first version of the Epic Sepsis Model (ESM v1), a proprietary penalized logistic regression model for sepsis prediction, which was implemented at hundreds of US hospitals. Despite its widespread deployment, this model was found to have poor discrimination and calibration in several external validations across different health systems.^12,13,14,15^ Specifically, the use of antibiotics as predictors, comparison against nonclinical outcome labels, and limited model generalizability may have contributed to poor performance and discrepancies between internal and external validations of the ESM v1.

In 2022, Epic Systems released a revised version of the sepsis prediction model (ESM v2), a gradient-boosted tree model that addressed prior methodological concerns and allowed for localized fine-tuning (ie, updating model parameters based on a site-specific historical dataset). In internal validation, Epic Systems has reported improved area under the receiver operating characteristic curve (AUROC) values for the ESM v2 between 0.83 and 0.86 across 3 internal validation sites (Tyler Sundberg and Elliot First, Epic Systems, Microsoft Teams meeting, February 25, 2025). As of August 2025, Epic Systems reports that 95 health care organizations (representing 731 individual hospitals) currently use this model for real-time sepsis prediction, while more than 100 organizations still use the original model. Despite broad adoption, an external multicenter validation of the new sepsis prediction model has yet to be reported.

Here we present a multicenter prospective validation of the ESM v2 for inpatient sepsis prediction at 4 major US health systems. Using real-time prospective data collected after implementation at each study site, we report on the performance of the new model against the original version, outline differences in performance across heterogeneous clinical sites, and compare model performance against clinician recognition of sepsis.

## Methods

This prognostic study was approved by the institutional review board at each participating site. The requirement for informed consent was waived due to a minimal risk exemption for secondary research. The reporting of this study was guided by Transparent Reporting of a Multivariable Prediction Model for Individual Prognosis or Diagnosis updated for artificial intelligence (TRIPOD+AI) reporting guidelines.^16^

### Study Setting

We conducted a prospective validation study at 4 large US health systems (University of Michigan, Ann Arbor, Michigan; Oregon Health & Science University, Portland, Oregon; Emory Healthcare, Atlanta, Geogia; and MetroHealth, Cleveland, Ohio). At each site, data were collected for a consecutive time period immediately following model implementation. Data collected at the University of Michigan and Oregon Health & Science University represented patients from a single large academic tertiary care center. Data collected at Emory Healthcare represented patients from a combination of 6 academic and community hospitals affiliated with Emory. Data collected at MetroHealth represented patients from a single large safety net hospital.

### Patient Population

All adult patients aged 18 years or older who were evaluated in the emergency department (ED) or were hospitalized with at least 1 prediction score from the new model and the original model were included in the study. Patients who met sepsis criteria, were discharged, or died before a model score was generated were excluded.

### Outcome Definition

Sepsis cases were defined using Sepsis-3 clinical criteria,^4^ which includes patients with evidence of organ dysfunction between 48 hours before and 24 hours after suspected infection. Organ dysfunction was defined as a 2-point or greater change in the Sequential Organ Failure Assessment (SOFA) score from encounter baseline. Suspected infection was defined as the cooccurrence of a body fluid culture and the first dose of at least 2 doses of antibiotics administered between 72 hours before up to 24 hours after the time of culture order. Model performance and lead time analysis was performed relative to the time of sepsis positivity, defined as the first time a patient met Sepsis-3 criteria within each hospital encounter. Additional details on our approach to sepsis labeling are available in the eMethods in Supplement 1.

### Sepsis Prediction Models

The sepsis prediction models generate scores between 0 and 100 that reflect a patient’s sepsis risk. Prediction scores for both models were calculated every 15 minutes on all ED and hospitalized patients. Predictions that occurred after the time of sepsis positivity were excluded. Before ESM v2 implementation, all study sites used the original model to trigger real-time sepsis alerts to health care practitioners. Following implementation of the new model, alerts were triggered based on ESM v2 scores and ESM v1 scores were calculated silently in the background. At all study sites, the new model was fine-tuned on a site-specific, historical training set of adult inpatients before implementation.

Per developer recommendation, ESM v2 prediction scores within the first hour of evaluation for patients without an available complete blood count (CBC) result were left-censored due to high variability observed in internal validation studies. Consequently, patients who met sepsis criteria within the first hour without a CBC result were excluded, as all ESM v2 scores within this time frame were censored. This first hour exclusion did not apply to the original, but these patients were also excluded from the model analysis for consistency. Additional details on the prediction models are available elsewhere^17^ and in the eMethods in Supplement 1.

### Statistical Analysis

Model discrimination was assessed using the area under the receiver operating curve (AUROC). To maintain consistency across sites and simulate a highly sensitive threshold, the 60% sensitivity threshold at each institution was used to calculate specificity, positive predictive value (PPV), negative predictive value (NPV), and the number needed to evaluate (NNE). We generated 95% CIs using 1000 bootstrap resamples. Model performance was analyzed at the encounter level and at the prediction level with 4-hour, 12-hour, and hospitalization-wide time horizons. Encounter-level analysis was based on the time of the first prediction score to exceed the threshold. Prediction-level performance was calculated using the same model thresholds that matched 60% sensitivity in the encounter-level analysis. Model calibration was evaluated using calibration plots. Analyses were performed separately at each site and results were pooled for comparison. No patient data were shared between sites. Additional notes on encounter-level vs prediction-level analysis are available in the eMethods in Supplement 1.

The timing of clinician sepsis recognition can affect the real-world performance and utility of sepsis prediction models.^15^ To measure model performance against clinician recognition of sepsis, we performed sensitivity analyses against the first indicators of clinician sepsis recognition (ie, earliest antibiotic order, lactate order, and body culture order), as well as a composite definition based on the earliest of any of these. The comparison event time was moved up from time of Sepsis-3 positivity to the time of the earliest clinical indicator of sepsis recognition, if present. Comparison against clinician recognition was performed at the encounter level.

A fairness audit was performed to determine if the model had variable performance across subpopulations stratified by age, sex, race, and ethnicity. Race and ethnicity were self-reported. Race was categorized as Asian, Black, White, other (defined as an inclusive category of any of the following: American Indian and Alaska Native, Asian Indian, Middle Eastern or North African, multiracial, Native Hawaiian or Pacific Islander, or other), and unknown. Ethnicity was categorized as Hispanic or non-Hispanic. For each subgroup, we report the baseline sepsis incidence rate, median and IQR of model scores and model AUROC.

To assess alert burden, we plotted the mean volume of prediction scores that exceeded the model alert threshold at each institution. To simulate alert burden within a hypothetical clinical workflow, we plotted the total alert volume using an 8-hour silencing strategy (ie, suppress subsequent alerts for 8 hours after an alert is raised). Analyses were conducted from July 23 to August 19, 2025, using Python software version 3.13 (Python Software Foundation).

## Results

### Cohort Demographics

A total of 227 091 inpatient encounters across the 4 health systems were assessed, including 32 642 encounters from the University of Michigan (November 11, 2024, to March 11, 2025), 36 211 encounters from the Oregon Health & Science University (August 31, 2023, to February 11, 2025), 54 420 encounters from Emory Healthcare (August 1, 2024, to September 30, 2024), and 103 818 encounters from MetroHealth (October 4, 2023, to October 23, 2024). Overall, 7401 encounters (3.3%; median [IQR] age, 65 [54-75] years; 3359 [45.4%] female; 198 Asian patients [2.7%]; 1824 Black patients [24.6%]; 4782 White patients [64.6%]; 526 Hispanic patients [7.1%]) met sepsis criteria and 221 695 encounters (96.7%; median [IQR] age [33-65] years; 123 563 [56.2%] female; 5495 Asian patients [2.5%] Asian; 85 319 Black patients [38.8%] Black; 109 039 White patients [49.6%] White; 21158 Hispanic patients [9.6%]) did not. An outline of patient demographics by study site is available in Table 1.

**Table 1.** Patient Characteristics by Hospital Encounter

Characteristic	Encounters, No. (%)											
	Michigan			OHSU			Emory			MetroHealth		
	Overall (n = 32 642)	No sepsis (n = 31 297)	Sepsis (n = 1345)	Overall (n = 36 211)	No sepsis (n = 33 885)	Sepsis (n = 2326)	Overall (n = 54 420)	No sepsis (n = 52 540)	Sepsis (n = 1880)	Overall (n = 103 818)	No sepsis (n = 101 968)	Sepsis (n = 1850)
Age, median (IQR), y	52 (33-68)	50 (32-68)	66 (54-74)	46 (33-66)	45 (33-65)	63 (49-73)	50 (35-67)	49 (34-67)	68 (55-78)	47 (33-63)	47 (33-63)	65 (55-75)
Sex												
Female	17 870 (55)	17 336 (55)	534 (40)	19 073 (53)	18 065 (53)	1008 (43)	31 258 (57)	30 337 (58)	921 (49)	58 721 (57)	57 825 (57)	896 (48)
Male	14 348 (44)	13 540 (43)	808 (60)	17 127 (47)	15 809 (47)	1318 (57)	23 153 (43)	22 194 (42)	959 (51)	45 076 (43)	44 122 (43)	954 (52)
Unknown	424 (1)	421 (1)	3 (<1)	11 (<1)	11 (<1)	0	9 (<1)	9 (<1)	0 (<1)	21 (<1)	21 (<1)	0
Race												
Asian	1129 (3)	1097 (4)	32 (2)	1432 (4)	1364 (4)	68 (3)	1932 (4)	1864 (4)	68 (4)	1200 (1)	1170 (1)	30 (2)
Black	4975 (15)	4838 (15)	137 (10)	1747(5)	1694 (5)	53 (2)	35 900 (66)	34 826 (66)	1074 (57)	44 521 (43)	43 961 (43)	560 (30)
White	23 761 (73)	22 683 (72)	1078 (80)	28 606 (79)	26 660 (79)	1946 (84)	12 866 (24)	12 237 (23)	629 (34)	48 588 (47)	47 459 (47)	1129 (61)
Other^a^	2260 (7)	2191 (7)	69 (5)	2181 (6)	2050 (6)	131 (6)	2192 (4)	2124 (4)	68 (4)	756 (1)	749 (1)	7 (<1)
Unknown	517 (2)	488 (2)	29 (2)	2245 (6)	2117 (6)	128 (6)	1530 (3)	1489 (3)	41 (2)	8753 (8)	8629 (8)	124 (7)
Ethnicity												
Hispanic	1728 (5)	1683 (5)	45 (3)	4677 (13)	4446 (13)	231 (10)	2904 (5)	2805 (5)	99 (5)	11 971 (12)	11 807 (12)	164 (9)
Non-Hispanic	30 158 (92)	28 913 (92)	1245 (93)	29 407 (81)	27 418 (81)	1989 (86)	49 417 (91)	47 709 (91)	1708 (91)	89 651 (86)	88 003 (86)	1648 (89)
Unknown	756 (2)	701 (2)	55 (4)	2127 (6)	2021 (6)	106 (5)	2099 (4)	2026 (4)	73 (4)	2196 (2)	2158 (2)	38 (2)
Illness complexity												
LOS, median (IQR), d	0.9 (0.2-3.0)	0.7 (0.2-2.6)	7.5 (4.3-13.2)	1.25 (0.3-4.3	1.0 (0.2-3.5)	11.1 (5.4-20.9)	0.2 (0.2-0.8)	0.2 (0.2-0.6)	6.0 (3.2-11.0)	0.2 (0.1-0.3)	0.2 (0.1-0.3)	7.1 (4.0-12.1)
In-hospital mortality	187 (0.6)	90 (0.3)	97 (7.2)	574 (1.6)	265 (0.8)	309 (12)	405 (0.7)	224 (0.4)	181 (9.6)	465 (0.4)	297 (0.3)	168 (9)
30-d mortality	338 (1.0)	205 (0.7)	133 (9.9)	797 (2.2)	467 (1.4)	330 (13.0)	541 (1.0)	321 (0.6)	220 (11.7)	798 (0.8)	575 (0.6)	223 (12)

Abbreviations: LOS, length of stay; OHSU, Oregon Health & Science University.

^a^ Other race was defined as an inclusive category of all patients labeled as any of the following under race: American Indian and Alaska Native, Asian Indian, Middle Eastern or North African, multiracial, Native Hawaiian or Pacific Islander, other.

### Sepsis Incidence

The baseline sepsis incidence ranged from 2.2% to 7.1% across sites, with higher sepsis rates observed at tertiary care centers (Table 2). Following implementation of the new model, sepsis incidence rates remained consistent with historical rates at all study sites. Median (IQR) time from hospital presentation to sepsis positivity ranged from 3.7 (1.8-9.3) hours to 10.3 (1.3-81.4) hours. The percentage of sepsis positive encounters excluded due to the first hour no CBC exclusion ranged from 9.8% to 30.1% across sites.

**Table 2.** Model Performance of the Epic Sepsis Model Version 2 Across Study Sites

Performance metric	Estimate (95% CI)			
	Michigan	OHSU	Emory	MetroHealth
Encounter-level prevalence, %				
Baseline sepsis incidence	5.9	7.1	3.7	2.2
First hour no CBC exclusion rate^a^	30.1	15.4	9.8	20.0
Postexclusion sepsis incidence	4.1	6.4	3.5	1.8
Sepsis cases with onset in ED	38.9	44.2	71.5	60.3
Median sepsis positivity, (IQR), h	8.7 (3.4-26.8)	7.2 (2.0-75.8)	3.7 (1.8-9.3)	4.9 (1.9-21.1)
60% Sensitivity Threshold Score	14	29	37	35
AUROC^b^	0.82 (0.81-0.83)	0.85 (0.85-0.86)	0.90 (0.90-0.91)	0.92 (0.92-0.93)
Sensitivity	0.60 (0.57-0.62)	0.60 (0.58-0.62)	0.60 (0.58-0.62)	0.60 (0.58-0.62)
Specificity	0.83 (0.83-0.84)	0.88 (0.88-0.89)	0.94 (0.93-0.94)	0.96 (0.96-0.96)
PPV	0.13 (0.13-0.14)	0.26 (0.25-0.27)	0.25 (0.24-0.26)	0.20 (0.19-0.21)
NPV	0.98 (0.98-0.98)	0.97 (0.97-0.97)	0.99 (0.98-0.99)	0.99 (0.99-0.99)
Prediction lead time to sepsis onset, median (IQR), h	7.3 (2.7-16.7)	10.3 (1.3-81.4)	1.9 (0.7-6.6)	4.2 (0.9-27.4)
Predictions per patient, median (IQR), No.	93 (19-299)	83 (18-402)	16 (9-30)	11 (6-28)
**4-h Time horizon**				
AUROC	0.75 (0.75-0.76)	0.82 (0.82-0.83)	0.86 (0.86-0.86)	0.86 (0.86-0.86)
Sensitivity	0.25 (0.24-0.26)	0.42 (0.41-0.43)	0.44 (0.43-0.44)	0.37 (0.37-0.38)
Specificity	0.96 (0.96-0.96)	0.93 (0.93-0.93)	0.97 (0.97-0.97)	0.96 (0.96, 0.96)
PPV	0.02 (0.01-0.02)	0.02 (0.02-0.02)	0.04 (0.04-0.04)	0.02 (0.02-0.02)
NNE, No.	69	60	24	43
**12-h Time horizon**				
AUROC	0.75 (0.74-0.75)	0.81 (0.81-0.82)	0.84 (0.83-0.84)	0.85 (0.85-0.85)
Sensitivity	0.22 (0.21-0.22)	0.41 (0.40-0.41)	0.42 (0.42-0.43)	0.36 (0.35-0.36)
Specificity	0.96 (0.96-0.96)	0.94 (0.94-0.94)	0.95 (0.95-0.95)	0.96 (0.96-0.96)
PPV	0.03 (0.03-0.03)	0.04 (0.04-0.04)	0.05 (0.05-0.05)	0.05 (0.05-0.05)
NNE, No.	35	26	22	21
**Hospitalization**				
AUROC	0.72 (0.72-0.73)	0.79 (0.79-0.79)	0.76 (0.76-0.77)	0.83 (0.83-0.83)
Sensitivity	0.19 (0.18-0.19)	0.31 (0.31-0.31)	0.24 (0.23-0.24)	0.31 (0.31-0.32)
Specificity	0.96 (0.96-0.96)	0.95 (0.95-0.95)	0.95 (0.95-0.95)	0.97 (0.97-0.97)
PPV	0.13 (0.12-0.13)	0.26 (0.26-0.26)	0.14 (0.14-0.14)	0.24 (0.24-0.24)
NNE, No.	8	4	7	4

Abbreviations: AUROC, area under the receiver operating characteristic curve; ED, emergency department; NNE, number needed to evaluate; NPV, negative predictive value; OHSU, Oregon Health & Science University; PPV, positive predictive value.

^a^ The first hour no CBC exclusion rate indicates the percentage of sepsis-positive encounters excluded from the study due to lack of an available CBC result in the first hour of evaluation, per Epic Sepsis Model Version 2 developer recommendations.

^b^ 95% CIs were generated with 1000 bootstrap resamples.

### Model Performance Across Study Sites

**Figure 1.**
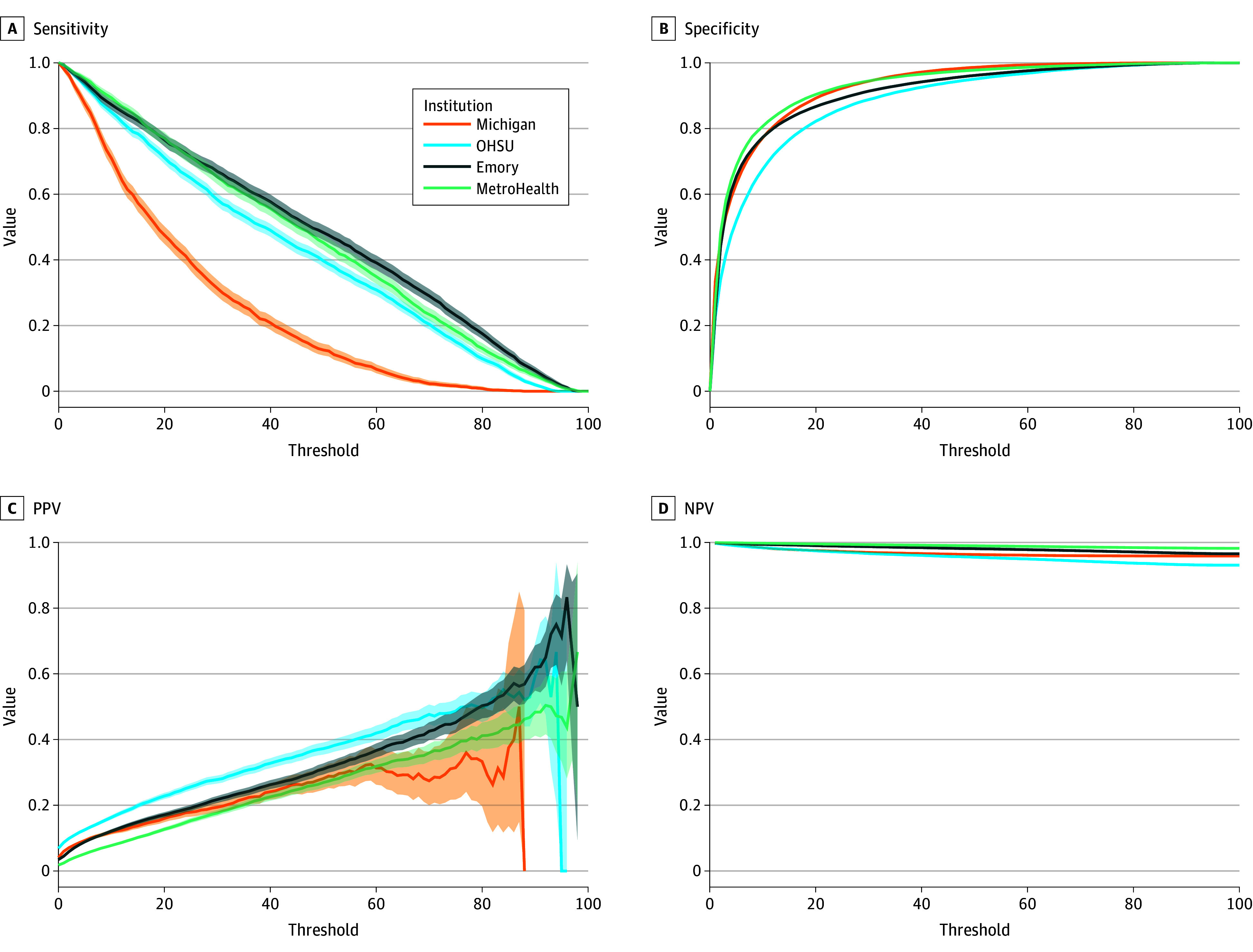
Encounter-Level Discriminative Performance of the Epic Sepsis Model Version 2 Across Study Sites Discriminative performance of the Epic Sepsis Model version 2 at the encounter-level was plotted across all possible model thresholds. The shaded region represents 95% confidence intervals for each performance statistic. NPV indicates negative predictive value; OHSU, Oregon Health & Sciences University; PPV, positive predictive value.

Performance of the new sepsis prediction model is outlined in Table 2. At the encounter level, the model had an AUROC ranging between 0.82 (95% CI, 0.81-0.83) and 0.92 (95% CI, 0.92-0.93). The model score threshold score matching 60% sensitivity ranged from 14 to 37 across institutions. At this threshold, specificity ranged from 0.83 (95% CI, 0.83-0.84) to 0.96 (95% CI, 0.96-0.96), PPV ranged from 0.13 (95% CI, 0.13-0.14) to 0.26 (95% CI, 0.25-0.27), and NPV ranged from 0.97 (95% CI, 0.97-0.97) to 0.99 (95% CI, 0.99-0.99). For true-positive cases, the median (IQR) prediction lead time before sepsis positivity ranged from 1.9 (0.7-6.6) hours to 10.3 (1.3-81.4) hours. Model performance across all possible thresholds is outlined in Figure 1. Calibration plots corresponding to model performance at each site are available in eFigures 1 to 4 in Supplement 1.

At the prediction level with a 4-hour time horizon, AUROC ranged from 0.75 (95% CI, 0.75-0.76) to 0.86 (95% CI, 0.86-0.86), sensitivity ranged from 0.25 (95% CI, 0.24-0.26) to 0.44 (95% CI, 0.43-0.44), specificity ranged from 0.93 (95% CI, 0.93-0.93) to 0.97 (95% CI, 0.97-0.97), PPV ranged from 0.02 (95% CI, 0.01-0.02) to 0.04 (95% CI, 0.04-0.04), and the NNE ranged from 24 to 69 (Table 2). AUROC dropped and PPV and NNE improved with longer time horizons.

### Model Performance Against Clinician Recognition

Performance of the new sepsis prediction model against clinician recognition of sepsis is summarized in Table 3. Comparison of the model against a composite of clinical indicators of clinician recognition (ie, antibiotics, lactate, and body culture orders) resulted in a small drop in discriminatory performance, with a resulting AUROC ranging from 0.80 (95% CI, 0.79-0.81) to 0.90 (95% CI, 0.89-0.90) across sites. For true-positive cases, the median (IQR) prediction lead time ahead of clinician recognition ranged from 1.4 (0.6-4.0) hours to 7.1 (2.4-15.1) hours.

**Table 3.** Performance of the Epic Sepsis Model Version 2 Against Clinician Recognition

Performance metric	Estimate (95% CI)			
	Michigan	OHSU	Emory	MetroHealth
AUROC^a^	0.82 (0.81-0.83)	0.85 (0.85-0.86)	0.90 (0.90-0.91)	0.92 (0.92-0.93)
AUROC vs antibiotic	0.81 (0.80-0.82)	0.84 (0.83-0.85)	0.90 (0.90-0.91)	0.92 (0.92-0.93)
AUROC vs lactate	0.81 (0.80-0.82)	0.83 (0.82-0.84)	0.90 (0.89-0.90)	0.90 (0.89-0.91)
AUROC vs body culture	0.82 (0.81-0.82)	0.83 (0.82-0.84)	0.90 (0.90-0.91)	0.91 (0.91-0.92)
AUROC vs clinician^b^	0.80 (0.79-0.81)	0.82 (0.82-0.83)	0.89 (0.88-0.89)	0.90 (0.89-0.90)
Prediction lead time to clinician recognition, median (IQR), h	7.1 (2.4-15.1)	3.4 (0.0-42.6)	1.4 (0.6-5.2)	3.2 (0.5-13.4)

Abbreviations: AUROC, area under the receiver operating characteristic curve; OHSU, Oregon Health & Science University.

^a^ 95% CIs were generated with 1000 bootstrap resamples.

^b^ AUROC vs clinician performance was calculated based on the earliest of antibiotic, lactate, or body culture orders.

### ESM v2 Performance Against ESM v1

**Figure 2.**
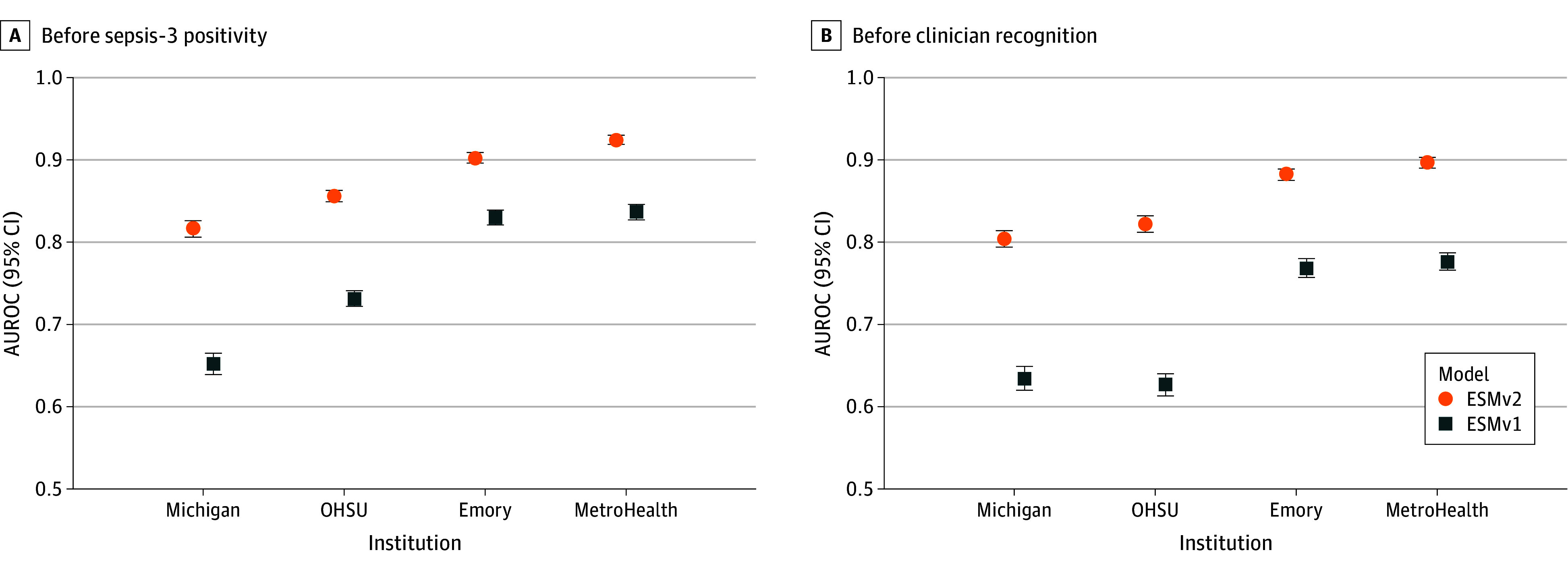
Dot Plot Showing Comparison of Epic Sepsis Model Version 2 and Version 1 Performance Before Sepsis-3 Positivity and Clinician Recognition Discriminative performance of the Epic Sepsis Model version 2 and version 1 before Sepsis-3 positivity and before clinician recognition were plotted. Red and blue markers indicate the area under the receiver operating characteristic curve (AUROC) of each corresponding model and error bars represent 95% CIs generated with 1000 bootstrap resamples. Clinician recognition of sepsis was defined as the time of the earliest antibiotic, lactate, or body culture order prior to Sepsis-3 positivity, if present, and defaulted to time of sepsis positivity otherwise. OHSU indicates Oregon Health & Sciences University.

A performance comparison between the new and original models before Sepsis-3 positivity and before clinician recognition is outlined in Figure 2. At the threshold matching a sensitivity of 0.60, the original had an encounter-level AUROC ranging from 0.65 (95% CI, 0.64-0.65) to 0.84 (95% CI, 0.83-0.85) and a PPV ranging from 0.07 (95% CI, 0.07-0.08) to 0.14 (95% CI, 0.14-0.15). The new model outperformed the ESM v1 across all performance metrics, at all model thresholds, and at all study sites. The new model also demonstrated better performance than the original when compared against clinician recognition of sepsis. A full overview of original model performance is available in eTable 1 in Supplement 1.

### Fairness Audit

The full results from the fairness audit for the new sepsis prediction model across all study sites are provided in eTables 2 to 5 in Supplement 1. Higher rates of sepsis were observed in older patients, male patients, White patients, and patients with non-Hispanic ethnicity. Model performance was closely associated with baseline sepsis rate. No major deviations from this trend were observed that suggested disproportionately better or worse performance based independently on age, sex, race, or ethnicity.

### Alert Burden

Plots of the mean volume of patient scores above the alert threshold and number of alerts raised with an 8-hour silencing strategy are available in eFigures 5 to 8 in Supplement 1. At all sites, a high frequency of alerts occurred immediately after admission, followed by a gradual decline as patients progressed further into their hospital course. A theoretical 8-hour silencing strategy substantially decreased total alert burden, but low PPV and high NNE persisted.

## Discussion

In this large multicenter prospective prognostic study, we found that the ESM v2 demonstrated moderate to strong discrimination for early sepsis prediction, with high variation in performance among institutions. The new sepsis prediction model achieved an overall encounter-level AUROC between 0.82 and 0.92 across sites, which is consistent with the performance independently reported by Epic Systems (AUROC, 0.83-0.86). The median prediction lead time to sepsis positivity (1.9-10.2 hours) suggested an adequate time window to reevaluate patients, order additional diagnostic testing, or initiate treatment. ESM v2 performance was superior to that of ESM v1 across all metrics. The new model also maintained a moderate level of performance when compared against clinician recognition of sepsis, where the original model did not. While the overall performance (eg, AUROC) of the new model was generally lower than that reported in other frontier sepsis prediction models, such as COMPOSER,^18^ Targeted Real-time Early Warning System,^19^ and SepsisWatch,^20^ it outperforms other more commonly deployed prediction models^21^ and clinical prediction scores, such as Systemic Inflammatory Response Syndrome, SOFA, and Quick SOFA.^22^ Furthermore, the new sepsis prediction model is currently deployed at hundreds of US hospitals, highlighting the importance of this and other external validation studies to verify its performance.

High variability was noted in model performance across study sites, highlighting the need for local validation prior to deployment. Discriminative accuracy (AUROC, 0.82-0.92), thresholds matching 60% sensitivity (14 to 37), prediction lead time (1.5-9.1 hours) and decrease in model performance against clinician suspicion all varied significantly across institutions. Several factors may have contributed to this. First, the new sepsis prediction model is likely more effective at identifying community-onset than hospital-onset sepsis. The new model demonstrated stronger performance at institutions with earlier sepsis onset times and a higher proportion of sepsis cases occurring in the ED, which is consistent with community-acquired cases. This is concordant with prior research suggesting that hospital-onset sepsis is more clinically complex and harder to predict than community-acquired sepsis.^23,24^ Second, the new model demonstrated weaker performance at sites with only tertiary care centers (University of Michigan and Oregon Health & Sciences University) than at sites that included community and safety net hospitals (Emory University and MetroHealth). Tertiary care sites had higher baseline sepsis rates, longer median times to sepsis positivity, longer median length of stay, a higher number of predictions per patient, and lower overall model performance against clinician recognition. These factors support prior evidence that patient complexity and baseline sepsis rates affect the performance of sepsis prediction models.^13^ Finally, the first hour CBC exclusion rate may have affected model performance differently across sites. Per developer guidelines, the new sepsis prediction model cannot produce accurate predictions in the first hour of admission in absence of a CBC result. Health systems differ in their ability to consistently draw and run a CBC within the first hour of care due to variations in clinical workflows, patient volume, and laboratory access. Institutions looking to implement this model should be cognizant of this model limitation, which can result in a substantial proportion of patients with sepsis (eg, up to 30% at the University of Michigan) not having any reliable model predictions prior to sepsis onset.

Despite improved performance, high alert burden remains a concern when implementing the new model. Our analysis found low PPV and high NNE associated with this model. For instance, NNE ranged from 21 to 35 at the 12-hour time horizon. This means that a health system using this model would need to manage or silence approximately 21 to 35 alerts to catch a single sepsis case of sepsis that occurs within the next 12 hours. The NNE improves to a range of 4 to 8 alerts at the hospitalization-wide time horizon, which can be helpful when considering encounter-level interventions, such as adding a patient to a sepsis watchlist or inpatient treatment pathway. The issue of high alert burden is not unique to this model and is a known limitation of all sepsis prediction models. The implementation of alert silencing strategies can help reduce total alert burden, but cannot improve alert accuracy. Clinical workflows, such as team huddles or bedside nurse screens, can help distinguish true positives from false positives and further guide effective sepsis care.

### Strengths and Limitations

This study had several strengths. First, this was the first large-scale multicenter validation study of this new sepsis prediction model with diverse representation across US geographic locations (Midwest, Pacific Northwest, South) and health system types (tertiary care center, community hospital, safety net hospital), allowing for a representative real-world evaluation of model performance. The standardized evaluation approach using Sepsis-3 criteria and a 60% sensitivity benchmark allowed for a clear comparison of performance between sites and models. Second, the use of prospective, real-time data collected immediately following implementation of the new model at each study site closely reflects model performance under real-world deployment conditions. This provides practical insights for health system leaders to guide expectations for model performance following implementation at their respective institutions. Furthermore, the analysis of model performance against multiple clinician recognition metrics and at multiple time horizons provides a nuanced perspective of the real-world clinical trade-offs among model precision, timeliness, and false-positive rates.

Despite these strengths, this study has several limitations. First, this study used Sepsis-3 clinical criteria to label sepsis cases, an approach that has imperfect concordance with the criterion standard approach of manual physician review.^25,26,27,28^ The use of Sepsis-3 abstraction for sepsis labeling allowed for a standardized approach to large-scale model validation across multiple study sites but limited the model performance analysis to clinician-centered outcomes, a known limitation with all forms of automated sepsis labeling. The Sepsis-3 consensus framework for sepsis coding and research recommends using concomitant orders for blood cultures and antibiotics within a specified period to define suspected infection.^4^ Use of this clinical sepsis definition limited the analysis to sepsis cases that eventually received cultures and antibiotic orders and did not include any sepsis cases that did not eventually receive treatment.

Second, model performance analysis was conducted after the new model was already integrated into clinical workflows. Changes in alert performance may have affected clinician behavior, including the frequency and timing of antibiotic or body culture orders. This may have introduced confirmation bias in our Sepsis-3 abstracted outcome labels and inflated model accuracy. Postimplementation model drift due to changes in clinician behavior is a widely recognized challenge in the evaluation of clinical prediction models with no standard solution.^29^ However, 2 factors in this study help mitigate these concerns. First, comparison of sepsis incidence rates following model integration against historical rates found no significant difference at our study sites, suggesting that introduction of the new model did not significantly change sepsis labeling rates. Second, all study sites previously used the original model for sepsis alerting, and no changes to the clinical workflow were made following implementation of the new model other than which model triggered sepsis alerts. Therefore, direct replacement of the original model with the new version was less likely to induce postimplementation model drift than the introduction of a novel system. While an ideal solution would have been simultaneous silent testing of both models, this was not feasible, as study sites were dependent on these models for clinical care.

Third, this study reported on model performance across the entire hospitalization and did not differentiate between sepsis diagnosed in the ED, on the inpatient floor, and in the intensive care unit. Prior sepsis research has suggested physiologic and epidemiologic heterogeneity between community-acquired and hospital-acquired sepsis populations^30^ and that sepsis prediction models may be more effective in identifying early-onset community-acquired sepsis cases in the ED than on the floor and intensive care unit.^31^ This is consistent with our findings that suggested superior performance of this model at sites with lower sepsis incidence rates and earlier times to sepsis positivity. Future research should assess the performance of the model against these and other subpopulations.

Fourth, because all our study sites performed model fine-tuning prior to implementation, we cannot comment on the performance of the base model without fine-tuning. Fifth, this study analyzed observational data collected immediately after implementation of the new model at each study site and was not designed to evaluate its impact on clinical outcomes, such as mortality. Additional research is needed to study the mortality impact of this sepsis prediction model.

## Conclusions

In this prognostic study of the ESM v2, our multicenter prospective validation performed across 4 large US health systems found improved model discrimination but high variability across institutions. Health systems seeking to implement this model should conduct internal evaluations to validate local performance, deploy alert silencing strategies, and design clinical workflows to help manage false positives.

## Notes

Supplement 1.
eMethods.
**eFigure 1.** Calibration Plot for the Epic Sepsis Model v2 at the University of Michigan **eFigure 2.** Calibration Plot for the Epic Sepsis Model v2 at Oregon Health & Science University **eFigure 3.** Calibration Plot for the Epic Sepsis Model v2 at Emory University **eFigure 4.** Calibration Plot for the Epic Sepsis Model v2 at MetroHealth **eTable 1.** Model Performance of the Epic Sepsis Model v1 Across Study Sites **eTable 2.** Fairness Audit of the ESM v2 Across Sub-Populations (Michigan) **eTable 3.** Fairness Audit of the ESM v2 Across Sub-Populations (OHSU) **eTable 4.** Fairness Audit of the ESM v2 Across Sub-Populations (Emory) **eTable 5.** Fairness Audit of the ESM v2 Across Sub-Populations (MetroHealth) **eFigure 5.** Unsilenced and Silenced Alert Frequency for the Epic Sepsis Model v2 at the University of Michigan **eFigure 6.** Unsilenced and Silenced Alert Frequency for the Epic Sepsis Model v2 at Oregon Health & Science University **eFigure 7.** Unsilenced and Silenced Alert Frequency for the Epic Sepsis Model v2 at Emory University **eFigure 8.** Unsilenced and Silenced Alert Frequency for the Epic Sepsis Model v2 at MetroHealth

Supplement 2.
Data Sharing Statement
