# Supplementary material for: Multicenter Prospective Validation of an Updated Proprietary Sepsis Prediction Model
Source: JAMA Netw Open. 2026 Feb 27;9(2):e260181. doi: 10.1001/jamanetworkopen.2026.0181 (PMC12949446; doi:10.1001/jamanetworkopen.2026.0181)
Supplement: Supplement 1. — eMethods. eFigure 1. Calibration Plot for the Epic Sepsis Model v2 at the University of Michigan eFigure 2. Calibration Plot for the Epic Sepsis Model v2 at Oregon Health & Science University eFigure 3. Calibration Plot for the Epic Sepsis Model v2 at Emory University eFigure 4. Calibration Plot for the Epic Sepsis Model v2 at MetroHealth eTable 1. Model Performance of the Epic Sepsis Model v1 Across Study Sites eTable 2. Fairness Audit of the ESM v2 Across Sub-Populations (Michigan) eTable 3. Fairness Audit of the ESM v2 Across Sub-Populations (OHSU) eTable 4. Fairness Audit of the ESM v2 Across Sub-Populations (Emory) eTable 5. Fairness Audit of the ESM v2 Across Sub-Populations (MetroHealth) eFigure 5. Unsilenced and Silenced Alert Frequency for the Epic Sepsis Model v2 at the University of Michigan eFigure 6. Unsilenced and Silenced Alert Frequency for the Epic Sepsis Model v2 at Oregon Health & Science University eFigure 7. Unsilenced and Silenced Alert Frequency for the Epic Sepsis Model v2 at Emory University eFigure 8. Unsilenced and Silenced Alert Frequency for the Epic Sepsis Model v2 at MetroHealth [file jamanetwopen-e260181-s001.pdf]

Supplemental Online Content

Wong A, Currey D, Schwinne M, et al. Multicenter prospective validation of an updated proprietary sepsis prediction model. *JAMA Netw Open*. 2026;9(2):e260181. doi:10.1001/jamanetworkopen.2026.0181

eMethods.

eFigure 1. Calibration Plot for the Epic Sepsis Model v2 at the University of Michigan

eFigure 2. Calibration Plot for the Epic Sepsis Model v2 at Oregon Health & Science University

eFigure 3. Calibration Plot for the Epic Sepsis Model v2 at Emory University

eFigure 4. Calibration Plot for the Epic Sepsis Model v2 at MetroHealth

eTable 1. Model Performance of the Epic Sepsis Model v1 Across Study Sites

eTable 2. Fairness Audit of the ESM v2 Across Sub-Populations (Michigan)

eTable 3. Fairness Audit of the ESM v2 Across Sub-Populations (OHSU)

eTable 4. Fairness Audit of the ESM v2 Across Sub-Populations (Emory)

eTable 5. Fairness Audit of the ESM v2 Across Sub-Populations (MetroHealth)

eFigure 5. Unsilenced and Silenced Alert Frequency for the Epic Sepsis Model v2 at the University of Michigan

eFigure 6. Unsilenced and Silenced Alert Frequency for the Epic Sepsis Model v2 at Oregon Health & Science University

eFigure 7. Unsilenced and Silenced Alert Frequency for the Epic Sepsis Model v2 at Emory University

eFigure 8. Unsilenced and Silenced Alert Frequency for the Epic Sepsis Model v2 at MetroHealth

This supplemental material has been provided by the authors to give readers additional information about their work.

© 2026 Wong A et al. *JAMA Network Open*.

eMethods.

Notes on the Sepsis-3 Outcome Definition

Sepsis cases were automated labeled using Sepsis-3 clinical criteria, defined as evidence of organ dysfunction between 48 hours before and 24 hours after suspected infection. Organ dysfunction was defined as a 2-point or greater change in the Sequential Organ Failure Assessment (SOFA) score from encounter baseline. Baseline SOFA scores were calculated using the most recent lab values and Glasglow Coma Scale available within the past year. Missing SOFA elements defaulted to a normal value if no information was documented. The PaO2 / FiO2 ratio was used to calculate respiratory SOFA scores, including estimates for FiO2, as configured within each organization. Mean arterial pressure (MAP) was calculated from systolic and diastolic blood pressures if a MAP value was not available. Suspected infection was defined as the co-occurrence of a body fluid culture and the first dose of at least two doses of antibiotics administered between 72 hours before up to 24 hours after the time of culture order. Patients who received only one dose of antibiotics within this 96-hour window were not included.

Rationale for Use of an Automated Sepsis-3 Outcome Definition

Sepsis cases were automatically labeled using Sepsis-3 clinical criteria for feasibility. While we acknowledge that automated sepsis labeling has been demonstrated to have imperfect concordance with gold standard physician review, this approach allowed us to maintain a uniform approach to sepsis labeling across all study sites for large patient populations, which would not have been feasible with manually validated outcome labels.

Notes on the Epic Sepsis Models

Both the ESM v2 and ESM v1 generate scores between 0 and 100 that reflect a patient's sepsis risk. Scores are unitless and thereby are not directly comparable on an absolute scale, but are comparable from a rank-ordering perspective. When implementing the ESM v2, each institution assigns a score threshold above which a sepsis alert will be triggered. The clinical workflow that occurs in response to an alert (e.g., EHR-based provider alert, team sepsis huddle) is also set by the individual institution.

Epic Sepsis Model v2 First Hour No CBC Exclusion

In their internal validation of the ESM v2, Epic Systems reported sporadic prediction scores for patients that do not have a CBC result available within the first hour of evaluation. Due to the inconsistency of these prediction scores, Epic recommends left-censoring of all ESM v2 prediction scores for patients that fall within these criteria. To remain aligned with developer guidelines and best capture real-world model performance, we excluded patients that fell within this first hour no CBC criteria from our study. To maintain consistency across analyses of the ESM v2 and the ESM v1, this cohort was also excluded when determining ESM v1 performance.

Epic Sepsis Model v2 Fine-Tuning

Prior to implementation, Epic Systems offers individual customers the option to fine-tune the ESM v2 on a local dataset of historical patients to improve performance. Fine-tuning is not required for implementation of the ESM v2, and institutions can instead choose to use baseline model weights if they opt out of model fine-tuning. Organizations with multiple hospitals can choose to perform separate fine-tuning for each site or combined fine-tuning using pooled data across all sites.

Epic Sepsis Model v2 Implementation Cost

Epic Systems contracts with individual customers to provide access to a suite of cloud-based predictive models, which includes the ESM v2. Once access to this suite of models is ascertained, there is no additional cost to fine-tuning or implementing the ESM v2.

Missing Values

© 2026 Wong A et al. *JAMA Network Open*.

The ESM v2 uses missingness as an additional piece of information in classification. For missing values among input features, the model assigns missing values to their own decision tree branch at each split, incorporating the fact that the value is missing into the prediction pathway. Missing values for outcome variables were imputed as normal (see above section “Additional Notes on the Sepsis-3 Outcome Definition”).

Statistical Significance

In this study, statistical significance between the performance of predictive models was determined by comparing 95% confidence intervals for the area under the receiver operating characteristic curve on the same test set.

Notes on Encounter-Level versus Prediction-Level Performance Analysis

Encounter-level and prediction-level consolidation are two unique approaches to the assessment of the discriminative accuracy of a clinical prediction model. We report both in our study to highlight different aspects of model performance.

For the encounter-level approach, each hospital encounter was treated as a single sample. All model predictions produced for the same hospital encounter were consolidated into a single prediction, which was positive if any single prediction score (prior to sepsis onset, if present) was positive, and negative only if all prediction scores were negative. In clinical terms, an encounter-level approach answers the question: *Was a sepsis alert ever raised for each patient during their hospitalization, and did it match whether the patient ended up having sepsis or not?*

For the prediction-level approach, each model prediction was treated as a separate sample. For each prediction, accuracy was determined by looking ahead to see if the patient eventually developed sepsis within the selected time horizon. For instance, using a 4-hour time horizon, a positive prediction would be labeled as correct if the patient developed sepsis within 4 hours immediately following the time of prediction. Using a hospitalization-level time horizon, a positive prediction would be labeled as correct if the patient ever developed sepsis during the remainder of the hospitalization. In clinical terms, a prediction-level approach answers the question: *How accurate is each individual model prediction at detecting the development of sepsis within the next X hours?*

Rationale for Focusing on Encounter-Level Performance

Both encounter-level and prediction-level analysis have their own strengths and weaknesses. With an encounter-level approach, a single false positive prediction can adversely affect model performance even if all other predictions in the same encounter were correctly negative. With a prediction-level approach, repeated subsequent positive predictions for the same high-risk patient are treated as equally important, even when repeated positive alerts have limited clinical utility once the initial risk for sepsis has already been identified.

In this study, we focus primarily on encounter-level performance because it most closely matches real-world clinical sepsis workflows. For instance, when considering clinical re-evaluation for a patient at risk for sepsis, the first positive model prediction is disproportionately the most important. A single positive prediction can trigger an interruptive EHR alert or activate a clinical huddle. Moreover, subsequent positive alerts provide little to no additional clinical utility and are often silenced to reduce alert burden and limit alert fatigue. Therefore, we centered our analysis on encounter-level model performance to best reflect how the ESM v2 may impact real-world clinical workflows. We nevertheless recognize the role of prediction-level analysis to comprehensively gauge model performance against other sepsis prediction systems, and we report these results alongside our encounter-level results.

© 2026 Wong A et al. *JAMA Network Open*.

eFigure 1. Calibration Plot for the Epic Sepsis Model v2 at the University of Michigan

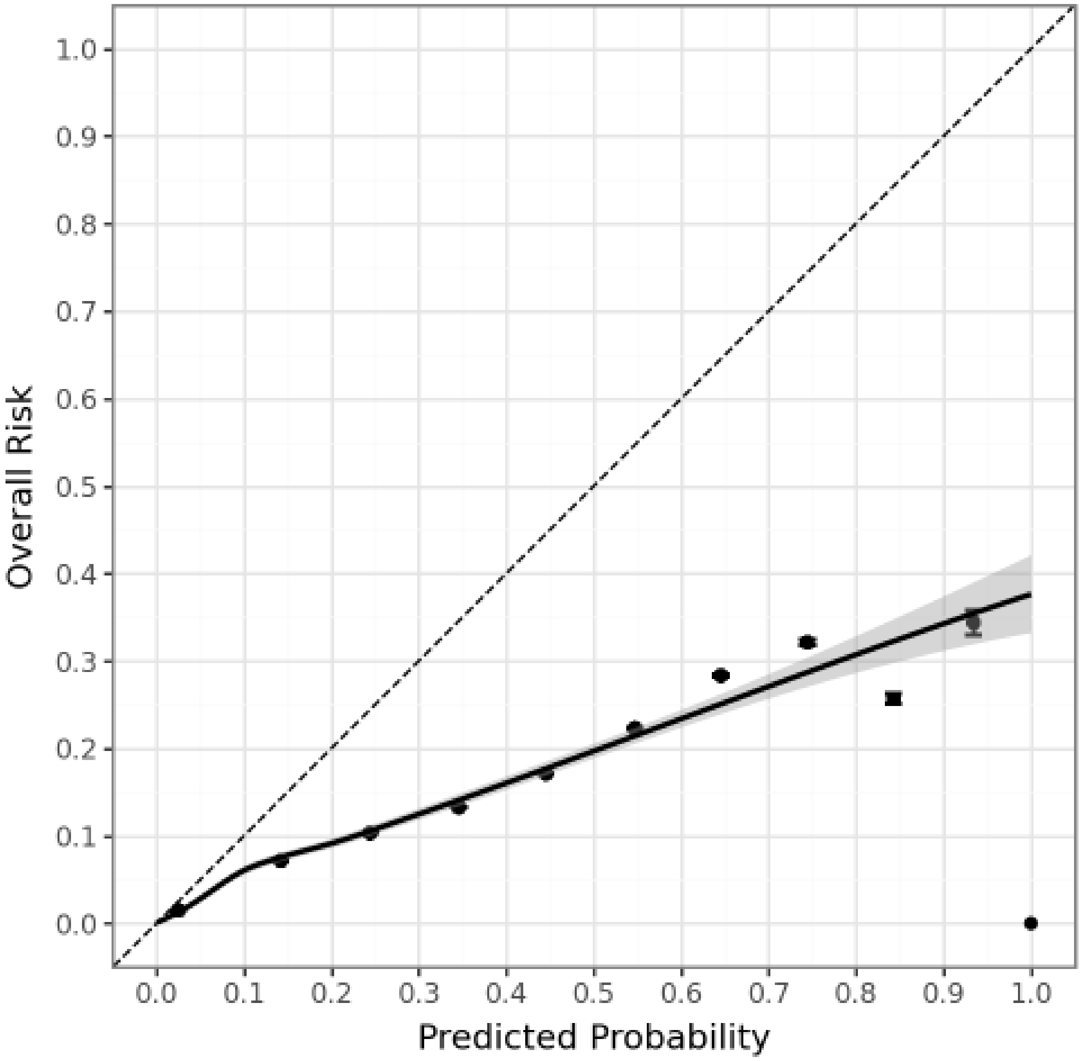

Calibration of Epic Sepsis Model v2 was plotted at the encounter level. The solid black line reflects non-parametric lowess curve. The shaded region represents 95% confidence.

© 2026 Wong A et al. *JAMA Network Open*.

eFigure 2. Calibration Plot for the Epic Sepsis Model v2 at Oregon Health & Science University

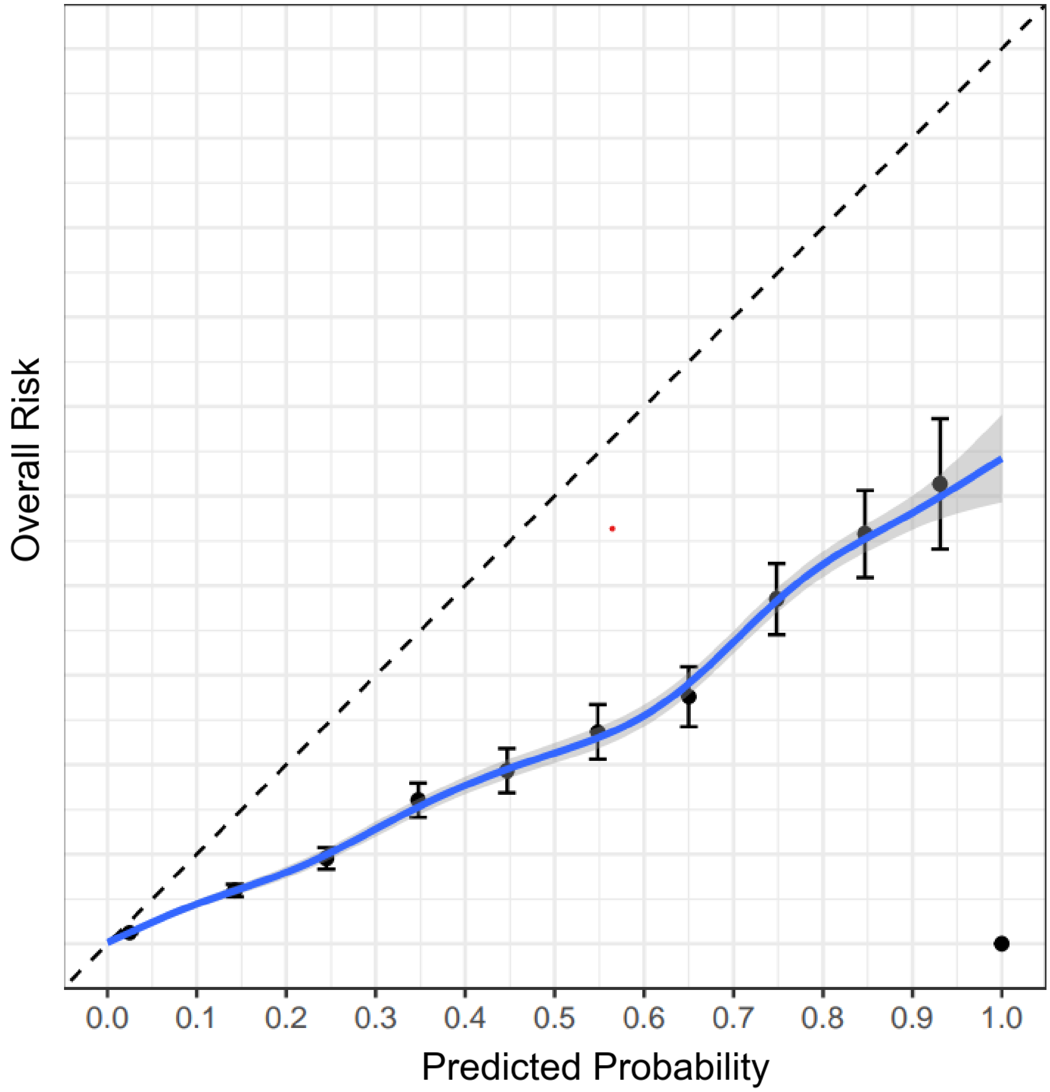

Calibration of Epic Sepsis Model v2 at the encounter level. The solid blue line reflects non-parametric lowess curve. The shaded region represents 95% confidence.

© 2026 Wong A et al. *JAMA Network Open*.

eFigure 3. Calibration Plot for the Epic Sepsis Model v2 at Emory University

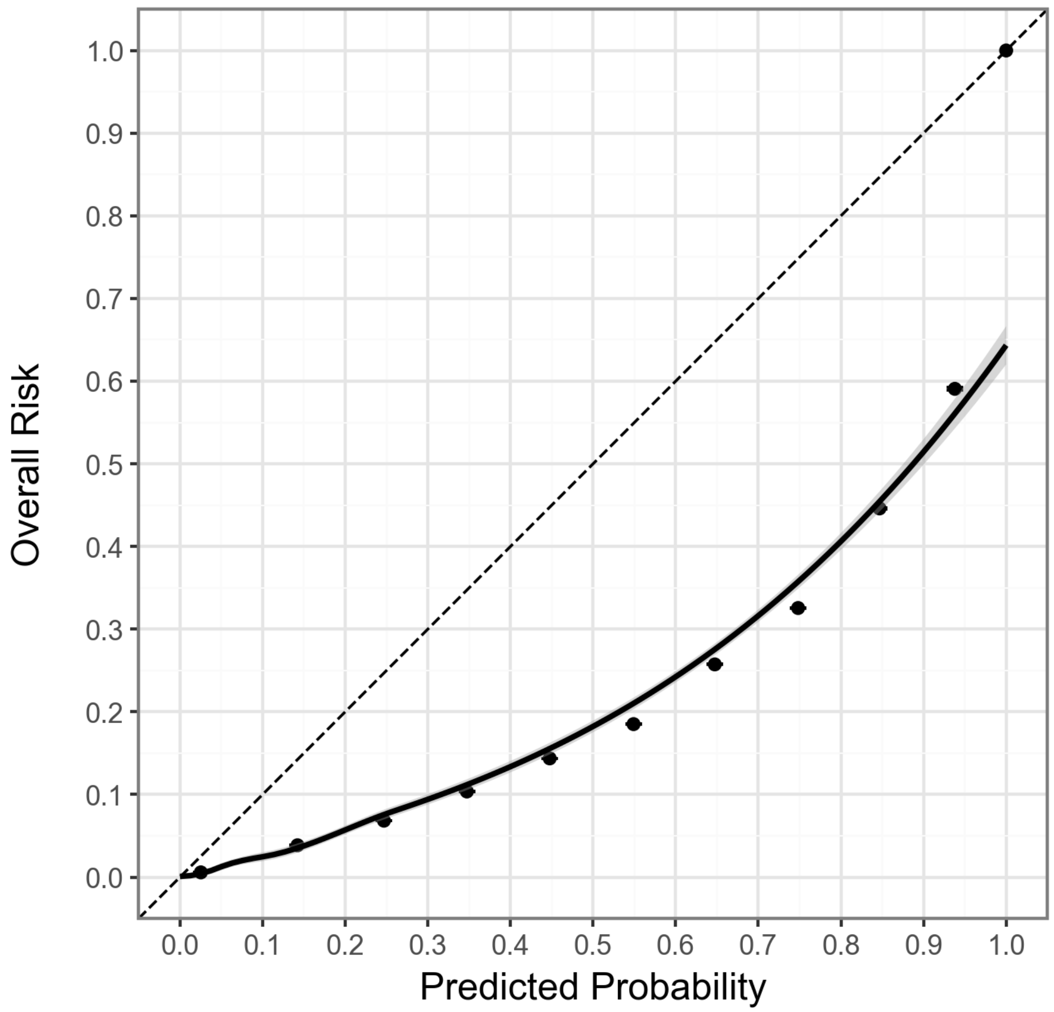

Calibration of Epic Sepsis Model v2 was plotted using 30,000 randomly selected samples at the encounter level. The Solid line reflects non-parametric lowess curve. The shaded region represents 95% confidence.

© 2026 Wong A et al. *JAMA Network Open*.

eFigure 4. Calibration Plot for the Epic Sepsis Model v2 at MetroHealth

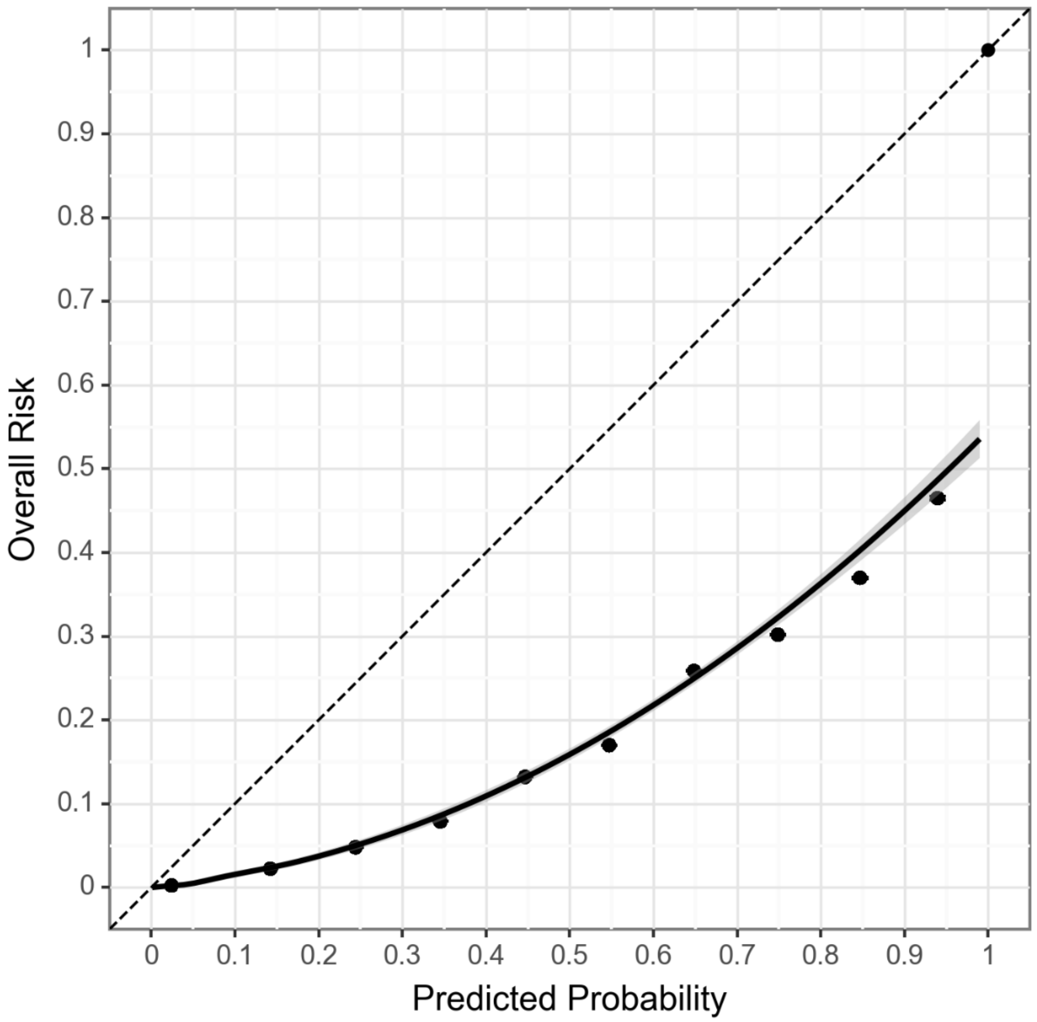

Calibration of Epic Sepsis Model v2 was plotted using 30,000 randomly selected samples at the encounter level. The Solid line reflects non-parametric lowess curve. The shaded region represents 95% confidence.

© 2026 Wong A et al. *JAMA Network Open*.

eTable 1. Model Performance of the Epic Sepsis Model v1 Across Study Sites

Performance Metric	Michigan	OHSU	Emory	MetroHealth
**Encounter-Level**				
Sepsis Incidence	5.9%	7.1%	3.5%	2.2%
Median Sepsis Onset, Hours (IQR)	8.7 (3.4-26.8)	7.2 (2.0-75.8)	3.7 (1.8-9.3)	4.9 (1.9-21.1)
AUROC (CI*)	0.65 (0.64-0.66)	0.73 (0.72-0.74)	0.83 (0.82-0.84)	0.84 (0.83-0.85)
Sensitivity (CI)	0.61 (0.58-0.63)	0.60 (0.58, 0.62)	0.60 (0.58-0.62)	0.60 (0.58-0.62)
Specificity (CI)	0.62 (0.62-0.63)	0.76 (0.75, 0.76)	0.86 (0.85-0.96)	0.88 (0.88-0.88)
PPV (CI)	0.07 (0.06-0.07)	0.14 (0.14-0.15)	0.13 (0.12-0.14)	0.08 (0.08-0.09)
NPV (CI)	0.97 (0.97-0.98)	0.97 (0.96-0.97)	0.98 (0.98-0.99)	0.99 (0.99-0.99)
**Prediction-Level**				
**4-Hour Time Horizon**				
AUROC (CI)	0.67 (0.67-0.68)	0.73 (0.73-0.73)	0.76 (0.76-0.76)	0.75 (0.75-0.76)
Sensitivity (CI)	0.34 (0.34-0.35)	0.32 0.32-0.33)	0.46 (0.46-0.47)	0.35 (0.34-0.35)
Specificity (CI)	0.83 (0.82-0.83)	0.91 (0.91-0.91)	0.86 (0.86-0.86)	0.92 (0.92-0.92)
PPV (CI)	0.005 (0.005-0.006)	0.01 (0.01-0.01)	0.01 (0.01-0.01)	0.01 (0.01-0.01)
NNE	182	105	91	91
**12-Hour**				
AUROC (CI)	0.64 (0.64-0.65)	0.70 (0.70-0.70)	0.72 (0.72-0.73)	0.74 (0.73-0.74)
Sensitivity (CI)	0.29 (0.29-0.30)	0.27 (0.27-0.28)	0.46 (0.45-0.46)	0.31 (0.31-0.32)
Specificity (CI)	0.83 (0.82-0.83)	0.91 (0.91-0.91)	0.81 (0.81-0.81)	0.92 (0.92-0.92)
PPV (CI)	0.01 (0.01-0.01)	0.02 (0.02-0.02)	0.02 (0.02-0.02)	0.02 (0.02-0.02)
NNE	96	53	67	48
**Hospitalization**				
AUROC (CI)	0.55 (0.55-0.55)	0.69 (0.69-0.69)	0.60 (0.60-0.60)	0.68 (0.68-0.68)
Sensitivity (CI)	0.23 (0.23-0.23)	0.21 (0.21-0.21)	0.32 (0.31-0.32)	0.23 (0.23-0.23)
Specificity (CI)	0.83 (0.82-0.83)	0.92 (0.92-0.92)	0.82 (0.82-0.82)	0.92 (0.92-0.92)
PPV (CI)	0.04 (0.04-0.04)	0.13 (0.12-0.13)	0.05 (0.05-0.05)	0.09 (0.09-0.09)
NNE	23	8	19	11

*95% confidence intervals were generated with 1,000 bootstrap resamples.

Abbreviations: AUROC indicates area under the receiver operating characteristic curve; CI, confidence interval; IQR, inter-quartile range; NNE, number needed to evaluate; NPV, negative predictive value; OHSU, Oregon Health & Science University; PPV, positive predictive value

© 2026 Wong A et al. *JAMA Network Open*.

eTable 2. Fairness Audit of the ESM v2 Across Sub-Populations (Michigan)

	Michigan (N = 32,642, ESM v2 Threshold = 14)			
	**Number of Patients (%)**	**Sepsis Rate**	**Median ESM v2 Score (IQR)**	**AUROC (95% CI*)**
**Age**				
18-29	6,417 (20)	1.0%	0.3 (0.1-1.1)	0.87 (0.83-0.91)
30-64	16,100 (49)	3.4%	1.2 (0.3-4.7)	0.82 (0.80-0.83)
65+	10,125 (31)	7.2%	6.5 (2.1-16.2)	0.74 (0.72-0.75)
**Sex**				
Female	17,870 (55)	3.0%	1.5 (0.4-6.1)	0.84 (0.82-0.86)
Male	14,348 (44)	5.6%	2.0 (0.4-9.9)	0.79 (0.78-0.80)
Unknown	424 (1)	0.7%	0.6 (0.2-1.9)	0.85 (0.66-0.85)
**Race**				
Asian	1129 (3)	2.8%	0.7 (0.2-3.5)	0.87 (0.81-0.92)
Black	4,975 (15)	2.8%	1.1 (0.3-5.0)	0.84 (0.81-0.87)
White	23,761 (73)	4.5%	2.0 (0.4-8.8)	0.80 (0.79-0.81)
Other	2,260 (7)	3.1%	0.8 (0.2-3.1)	0.87 (0.83-0.92)
Unknown	517 (2)	5.6%	1.1 (0.2-6.6)	0.84 (0.77-0.92)
**Ethnicity**				
Hispanic	1728 (5)	2.6%	0.7 (0.2-3.2)	0.87 (0.92-0.93)
Non-Hispanic	30,158 (92)	4.1%	1.7 (0.4-7.7)	0.81 (0.80-0.82)
Unknown	756 (2)	7.3%	1.8 (0.3-9.5)	0.82 (0.78-0.87)

*95% confidence intervals were generated with 1000 bootstrap resamples.

Abbreviations: AUROC indicates area under the receiver operating characteristic curve; CI, confidence interval; ESM, Epic Sepsis Model; IQR, interquartile range.

© 2026 Wong A et al. *JAMA Network Open*.

eTable 3. Fairness Audit of the ESM v2 Across Sub-Populations (OHSU)

OHSU (N = 36,394, ESM v2 Threshold = 30)				
	**Number of Patients (%)**	**Sepsis Rate**	**Median ESM v2 Score (IQR)**	**AUROC (95% CI*)**
**Age**				
18-29	6,437 (18)	2.5%	1.7 (0.4-6.0)	0.91 (0.89-0.93)
30-64	20,081 (55)	6.0%	4.2 (1.1-13.3)	0.86 (0.85-0.87)
65+	9,876 (27)	11.6%	13.1 (5.0-29.8)	0.77 (0.76-0.79)
**Sex**				
Female	19,143 (53)	5.6%	4.3 (1.2-13.4)	0.859 (0.848-0.870)
Male	17,240 (47)	8.3%	6.6 (1.4-20.9)	0.840 (0.829-0.850)
Unknown	11 (<0.1)	0.0%	3.0 (0.8-20.5)	N/A
**Race**				
Asian	1,440 (4)	5.3%	4.9 (1.3-14.4)	0.88 (0.84-0.91)
Black	1,755 (5)	3.5%	2.4 (0.5-9.0)	0.89 (0.86-0.93)
White	28,759 (80)	7.3%	5.7 (1.4-17.7)	0.85 (0.84-0.86)
Other	2,189 (6)	6.3%	4.2 (1.1-14.4)	0.84 (0.81-0.88)
Unknown	2,251 (6)	6.0%	3.8 (0.9-13.7)	0.86 (0.83-0.89)
**Ethnicity**				
Hispanic	4,699 (13)	5.4%	3.5 (0.9-12.0)	0.89 (0.87-0.91)
Non-Hispanic	29,562 (81)	7.3%	5.7 (1.4-17.6)	0.85 (0.84-0.85)
Unknown	2,133 (6)	5.3%	3.9 (1.0-14.7)	0.86 (0.83-0.89)

*95% confidence intervals were generated with 1000 bootstrap resamples.

Abbreviations: AUROC indicates area under the receiver operating characteristic curve; CI, confidence interval; ESM, Epic Sepsis Model; IQR, interquartile range; OHSU, Oregon Health & Science University.

© 2026 Wong A et al. *JAMA Network Open*.

eTable 4. Fairness Audit of the ESM v2 Across Sub-Populations (Emory)

Emory (N = 54,420, ESM v2 Threshold = 37)				
	**Number of Patients (%)**	**Sepsis Rate**	**Median ESM v2 Score (IQR)**	**AUROC (95% CI*)**
**Age**				
18-29	8,897 (16)	0.7%	0.9 (0.6-2.1)	0.90 (0.85-0.95)
30-64	30,195 (56)	2.5%	2.0 (0.9-6.0)	0.90 (0.89-0.92)
65+	15,328 (28)	7.0%	9.9 (3.6-28.3)	0.85 (0.84-0.86)
**Sex**				
Female	31,258 (57)	2.9%	2.6 (1.0-9.1)	0.91 (0.90-0.92)
Male	23,153 (43)	4.1%	2.7 (1.0-11.1)	0.89 (0.88-0.90)
Unknown	9 (0)	0.0%	4.0 (2.2-8.5)	N/A
**Race**				
Asian	1,932 (4)	3.5%	3.4 (1.2-11.6)	0.87 (0.83-0.91)
Black	35,900 (66)	3.0%	2.2 (0.9-7.8)	0.91 (0.91-0.92)
White	12,866 (24)	4.9%	4.9 (1.6-17.6)	0.87 (0.85-0.88)
Other	2,192 (4)	3.1%	1.9 (0.8-6.6)	0.91 (0.87-0.94)
Unknown	1,530 (3)	2.7%	2.3 (1.0-7.5)	0.96 (0.94-0.97)
**Ethnicity**				
Hispanic	2,904 (5)	3.4%	2.1 (0.9-7.4)	0.89 (0.86-0.93)
Non-Hispanic	49,417 (91)	3.5%	2.7 (1.0-10.1)	0.90 (0.90-0.91)
Unknown	2,099 (4)	3.5%	2.6 (1.1-8.6)	0.93 (0.90-0.96)

*95% confidence intervals were generated with 1000 bootstrap resamples.

Abbreviations: AUROC indicates area under the receiver operating characteristic curve; CI, confidence interval; ESM, Epic Sepsis Model; IQR, interquartile range.

© 2026 Wong A et al. *JAMA Network Open*.

eTable 5. Fairness Audit of the ESM v2 Across Sub-Populations (MetroHealth)

MetroHealth (N = 103,818, ESM v2 Threshold = 35)				
	**Number of Patients (%)**	**Sepsis Rate**	**Median ESM v2 Score (IQR)**	**AUROC (95% CI*)**
**Age**				
18-29	20,235 (19)	0.3%	0.8 (0.5-2.2)	0.91 (0.86-0.95)
30-64	60,902 (59)	1.4%	2.1 (1.0-6.5)	0.93 (0.92-0.94)
65+	22,681 (22)	4.1%	6.3 (2.1-19.5)	0.87 (0.86-0.88)
**Sex**				
Female	58,721 (57)	1.5%	2.1 (0.9-6.8)	0.92 (0.91-0.93)
Male	45,076 (43)	2.1%	2.2 (0.9-8.3)	0.92 (0.92-0.93)
Unknown	21 (0)	0.0%	0.8 (0.3-20.4)	N/A
**Race**				
Asian	1,200 (1)	2.5%	2.3 (1.0-8.1)	0.93 (0.89-0.96)
Black	44,521 (43)	1.3%	1.8 (0.8-5.7)	0.93 (0.92-0.94)
White	48,588 (47)	2.3%	2.7 (0.1-9.7)	0.91 (0.91-0.92)
Other	756 (1)	0.9%	2.0 (0.9-6.1)	0.97 (0.95-0.99)
Unknown	8,753 (8)	1.4%	1.9 (0.8-6.2)	0.94 (0.92-0.96)
**Ethnicity**				
Hispanic	11,971 (12)	1.4%	1.9 (0.8-6.0)	0.92 (0.90-0.94)
Non-Hispanic	89,651 (86)	1.8%	2.2 (0.9-7.5)	0.92 (0.92-0.93)
Unknown	2,196 (2)	1.7%	2.2 (0.9-7.9)	0.96 (0.94-0.98)

*95% confidence intervals were generated with 1000 bootstrap resamples.

Abbreviations: AUROC indicates area under the receiver operating characteristic curve; CI, confidence interval; ESM, Epic Sepsis Model; IQR, interquartile range.

© 2026 Wong A et al. *JAMA Network Open*.

eFigure 5. Unsilenced and Silenced Alert Frequency for the Epic Sepsis Model v2 at the University of Michigan

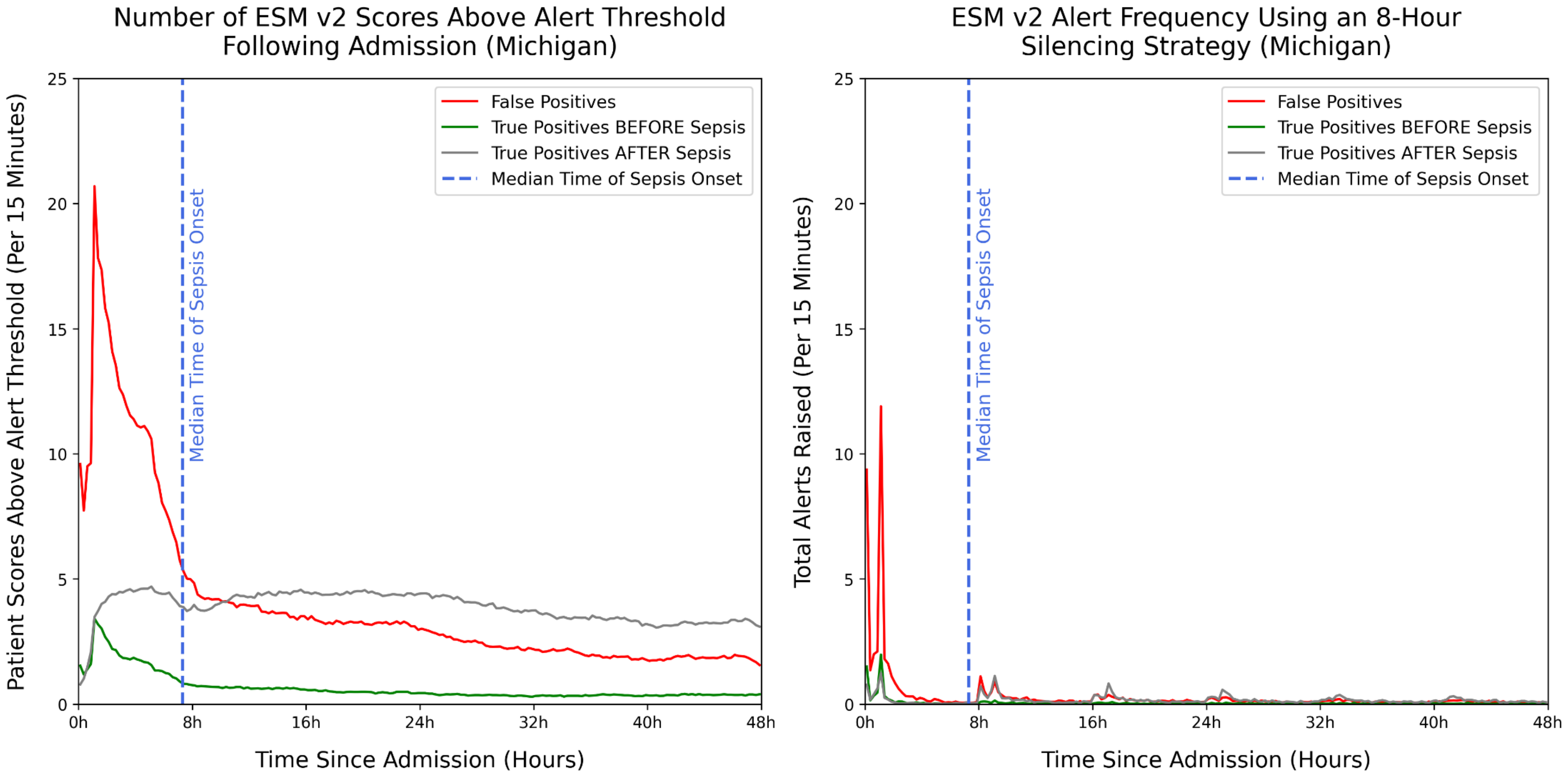

Alert frequency from the Epic Sepsis Model v2 was graphed as a function of admission time from the University of Michigan (800-bed tertiary care academic hospital). The graph on the left represents the number of patient scores that fall above the alert threshold on a 15-minute basis, representing the theoretical number of alerts that would be raised if no silencing strategy was employed. The graph on the right represents the number of alerts that would be raised if an 8-hour silencing strategy is employed (all subsequent alerts for the next 8 hours are silenced after an initial alert is raised). The red line represents false positive alerts, the green line true positive alerts that occur prior to sepsis onset, and the grey line true positive alerts that occur after sepsis onset. The dashed blue line reflects the median time of sepsis onset at the University of Michigan.

© 2026 Wong A et al. *JAMA Network Open*.

eFigure 6. Unsilenced and Silenced Alert Frequency for the Epic Sepsis Model v2 at Oregon Health & Science University

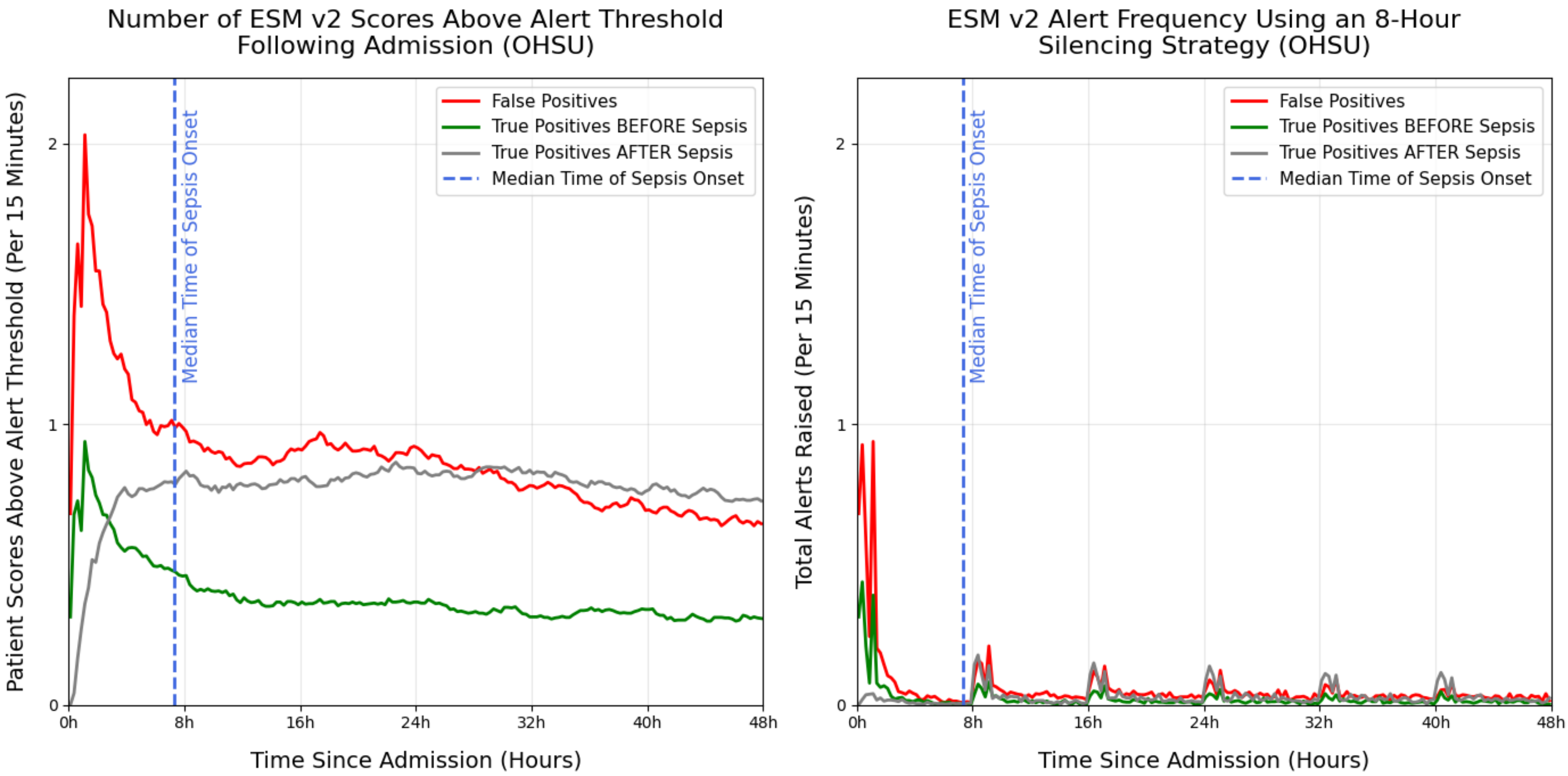

Alert frequency from the Epic Sepsis Model v2 was graphed as a function of admission time from Oregon Health & Science University (OHSU; 550-bed tertiary care academic hospital). The graph on the left represents the number of patient scores that fall above the alert threshold on a 15-minute basis, representing the theoretical number of alerts that would be raised if no silencing strategy was employed. The graph on the right represents the number of alerts that would be raised if an 8-hour silencing strategy is employed (all subsequent alerts for the next 8 hours are silenced after an initial alert is raised). The red line represents false positive alerts, the green line true positive alerts that occur prior to sepsis onset, and the grey line true positive alerts that occur after sepsis onset. The dashed blue line reflects the median time of sepsis onset at OHSU.

© 2026 Wong A et al. *JAMA Network Open*.

eFigure 7. Unsilenced and Silenced Alert Frequency for the Epic Sepsis Model v2 at Emory University

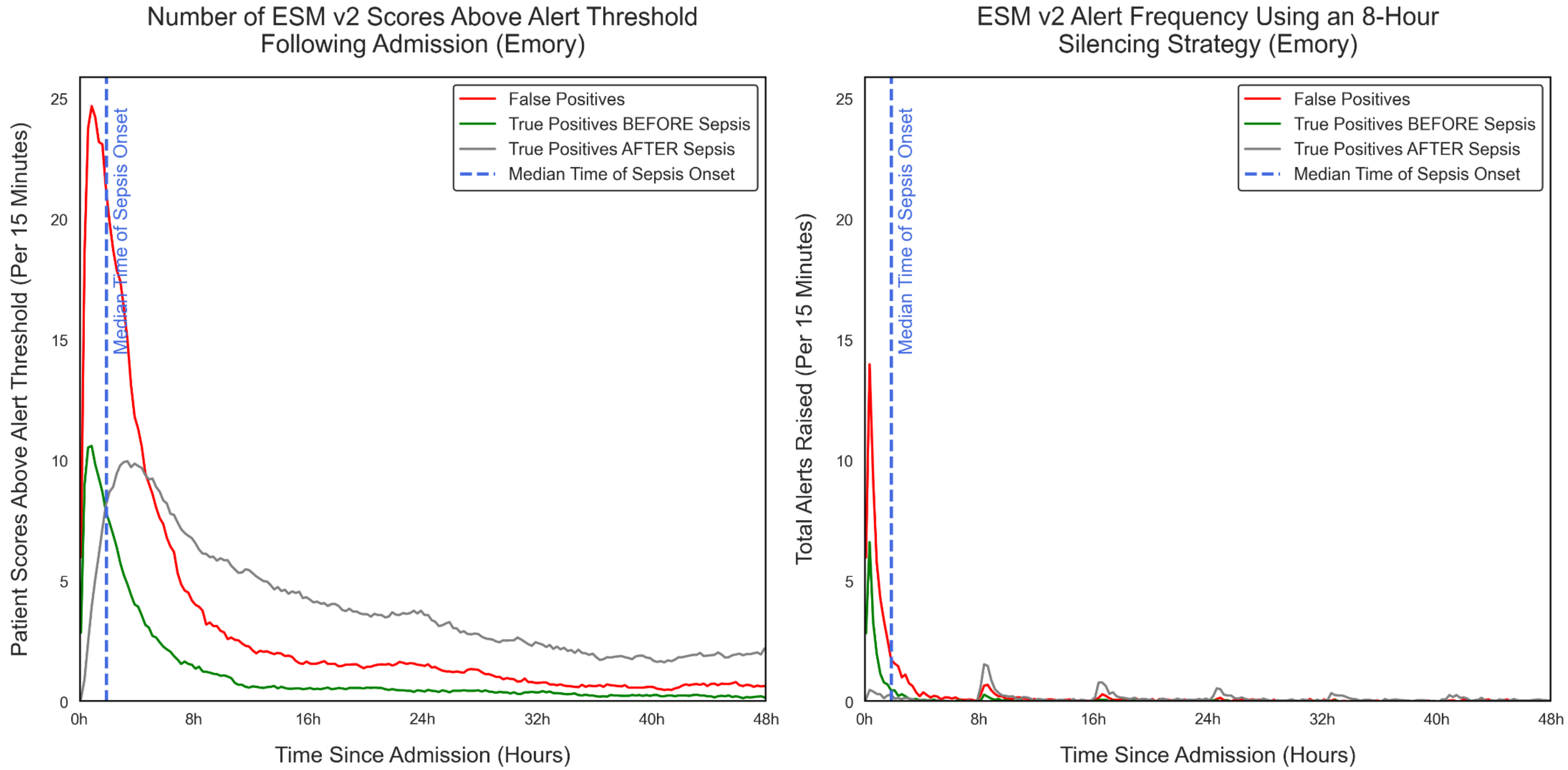

Alert frequency from the Epic Sepsis Model v2 was graphed as a function of admission time from 6 affiliated Emory University hospitals (combination of approximately 2,300 beds across both academic and community hospitals). The graph on the left represents the number of patient scores that fall above the alert threshold on a 15-minute basis, representing the theoretical number of alerts that would be raised if no silencing strategy was employed. The graph on the right represents the number of alerts that would be raised if an 8-hour silencing strategy is employed (all subsequent alerts for the next 8 hours are silenced after an initial alert is raised). The red line represents false positive alerts, the green line true positive alerts that occur prior to sepsis onset, and the grey line true positive alerts that occur after sepsis onset. The dashed blue line reflects the median time of sepsis onset at Emory.

© 2026 Wong A et al. *JAMA Network Open*.

eFigure 8. Unsilenced and Silenced Alert Frequency for the Epic Sepsis Model v2 at MetroHealth

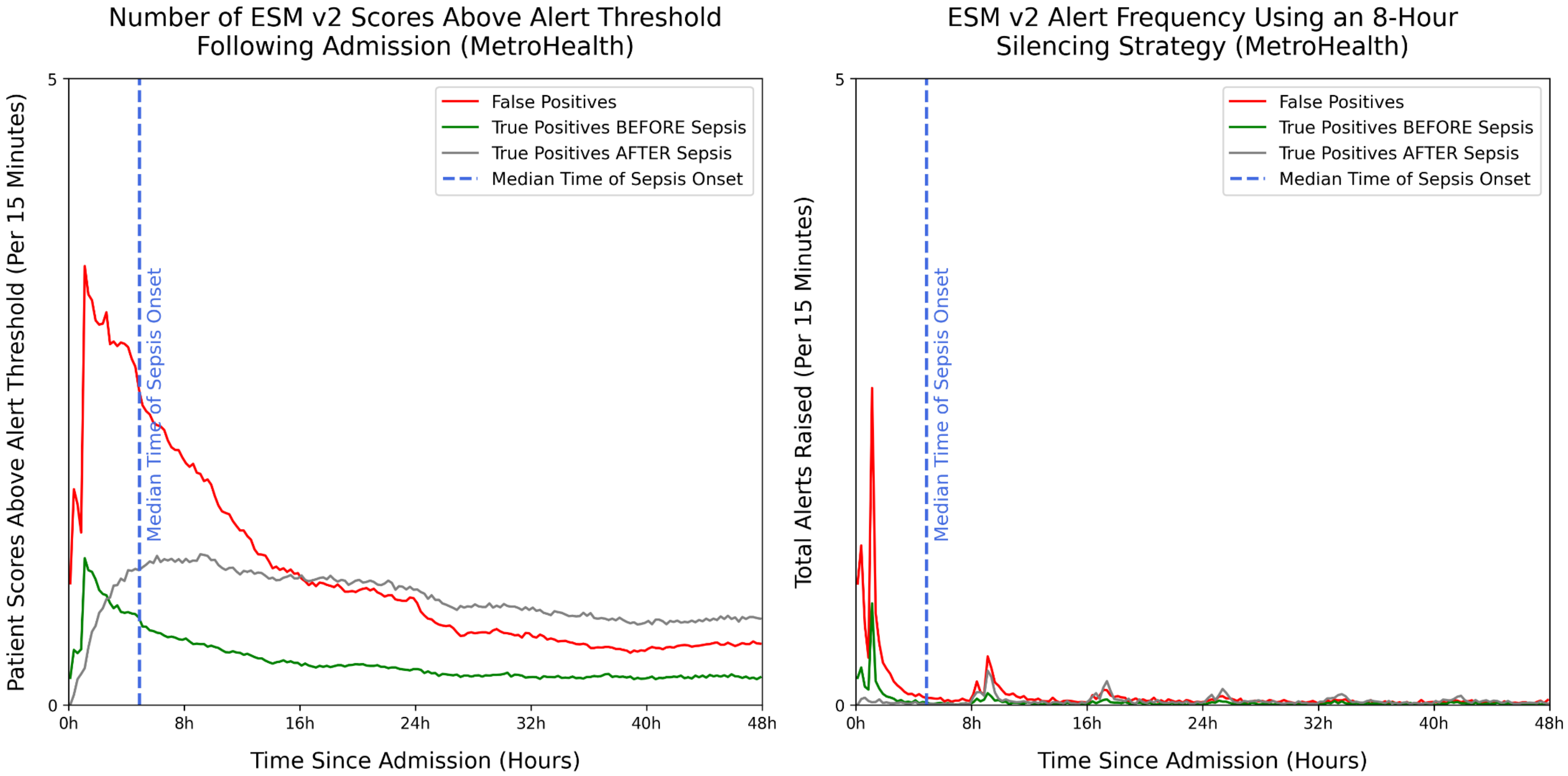

Alert frequency from the Epic Sepsis Model v2 was graphed as a function of admission time from MetroHealth (700-bed safety net hospital). The graph on the left represents the number of patient scores that fall above the alert threshold on a 15-minute basis, representing the theoretical number of alerts that would be raised if no silencing strategy was employed. The graph on the right represents the number of alerts that would be raised if an 8-hour silencing strategy is employed (all subsequent alerts for the next 8 hours are silenced after an initial alert is raised). The red line represents false positive alerts, the green line true positive alerts that occur prior to sepsis onset, and the grey line true positive alerts that occur after sepsis onset. The dashed blue line reflects the median time of sepsis onset at MetroHealth.

© 2026 Wong A et al. *JAMA Network Open*.
