# Supplementary material for: Multicenter Prospective Validation of an Updated Proprietary Sepsis Prediction Model
Source: JAMA Netw Open. 2026 Feb 27;9(2):e260181. doi: 10.1001/jamanetworkopen.2026.0181 (PMC12949446; doi:10.1001/jamanetworkopen.2026.0181)
Supplement: Supplement 2. — Data Sharing Statement [file jamanetwopen-e260181-s002.pdf]

Data Sharing Statement

Wong. Multicenter Prospective Validation of an Updated Proprietary Sepsis Prediction Model. *JAMA Netw Open*. Published February 27, 2026. doi:10.1001/jamanetworkopen.2026.0181

Data
Data available: No

Additional Information
Explanation for why data not available: The data used in this study are protected health information and will not be made publicly available.
